# Animal Models of Hyperoxaluria and Their Relevance for Fundamental Research and Clinical Practice

**DOI:** 10.3390/ijms27177885

**Published:** 2026-09-03

**Authors:** Dominika Szkopek-Zaworska, Mariusz Strutyński, Janine Donaldson, Stefan Pierzynowski, Kateryna Pierzynowska, Tomasz Jacek

**Affiliations:** 1 Large Animal Models Laboratory, The Kielanowski Institute of Animal Physiology and Nutrition, Polish Academy of Sciences, 05-110 Jabłonna, Poland; 2 Department of Animal Physiology, The Kielanowski Institute of Animal Physiology and Nutrition, Polish Academy of Sciences, 05-110 Jabłonna, Poland; kateryna.pierzynowska@biol.lu.se; 3 National Research Institute of Animal Production, 32-083 Balice, Poland; 4 Anara AB, 23132 Trelleborg, Sweden; mariuszstrutynski@lekam.pl (M.S.); janine.donaldson@wits.ac.za (J.D.); stefan.pierzynowski@biol.lu.se (S.P.); 5 Department of Physiology, School of Biomedical Sciences, Faculty of Health Sciences, University of the Witwatersrand, Johannesburg 2193, South Africa; 6 Department of Biology, Lund University, 22362 Lund, Sweden; 7 Department of Medical Biology, Institute of Rural Health, 20-090 Lublin, Poland

**Keywords:** hyperoxaluria, calcium oxalate, nephrolithiasis, animal models, oxalate decarboxylase

## Abstract

In vivo models have been central to understanding hyperoxaluria pathogenesis and to the development of emerging therapeutic strategies, but no single model fully reproduces the complexity of human disease. This narrative review critically evaluates currently available in vivo models of hyperoxaluria and provides a framework for their selection, interpretation, and integration according to the specific phenotype and biological or therapeutic question under investigation. A structured, non-systematic literature search was conducted in PubMed, Web of Science, Scopus, and Google Scholar, with emphasis on peer-reviewed primary studies and relevant reviews addressing experimental models, disease mechanisms, and therapeutic interventions. Chemically and dietary induced, genetic, enteric and microbial, and large-animal models were evaluated with respect to model validity, experimental endpoints, pathophysiological relevance, and translational potential. A central feature of this review is the distinction between related but non-interchangeable phenotypes, including hyperoxaluria, crystalluria, nephrocalcinosis, oxalate nephropathy, and nephrolithiasis. This distinction provides the conceptual basis for comparing models that reproduce different stages or consequences of oxalate exposure rather than treating all calcium oxalate-associated phenotypes as equivalent. Chemically induced models are particularly useful for studying hyperoxaluria, calcium oxalate crystallization, and acute or subacute renal injury, whereas genetic models reproduce specific molecular defects underlying primary hyperoxaluria and support the development of mechanism-based therapies. Enteric and microbial models address intestinal oxalate handling and the gut–kidney axis and are particularly relevant for investigating gut-directed therapeutic strategies. Naturally occurring models may provide complementary insight into chronic clinical phenotypes, whereas large-animal models can facilitate selected translational and experimentally intensive investigations. The reviewed evidence demonstrates that no model can be considered superior. Instead, model selection should be guided by the specific mechanism, phenotype, or therapeutic intervention under investigation. This review therefore proposes a phenotype-oriented and question-driven framework for model selection and interpretation, taking into consideration validity, reproducibility, ethical aspects, and the limits of translational extrapolation.

## 1. Introduction

Oxalate is a final metabolic end-product in humans that undergoes no further metabolism or conjugation and is eliminated predominantly through renal excretion. Its urinary excretion reflects the balance of three major sources: endogenous hepatic synthesis from glyoxylate and glycolate, intestinal absorption of dietary oxalate, and, under pathological conditions, altered renal and intestinal handling of oxalate [1]. Because oxalate is a divalent anion, with a high affinity for calcium, even relatively small increases in urinary oxalate concentration can markedly increase calcium oxalate (CaOx) supersaturation, making hyperoxaluria one of the strongest urinary determinants of CaOx stone formation [2]. CaOx stones account for approximately 70–80% of kidney stones, and persistent hyperoxaluria promotes tubular crystal retention, epithelial injury, interstitial inflammation, and progressive nephron loss. Consequently, the clinical spectrum of hyperoxaluria extends beyond nephrolithiasis to include nephrocalcinosis, acute and chronic oxalate nephropathy, chronic kidney disease (CKD), and, in its most severe form, systemic oxalosis [3,4]. Kidney stone disease is common, and its prevalence continues to increase with occurrence rates ranging between 7–13% in North America [5], 5–9% in Europe, and 1–5% in Asia [6]. Thus, even the diet-associated, common forms of hyperoxaluria represent a substantial and growing public-health burden.

Hyperoxaluria is traditionally classified into primary and secondary forms. Primary hyperoxalurias (PH1–PH3) are autosomal-recessive disorders of glyoxylate metabolism, caused by defects in alanine–glyoxylate aminotransferase (AGT, encoded by *AGXT*; PH1), glyoxylate/hydroxypyruvate reductase (GRHPR; PH2), or 4-hydroxy-2-oxoglutarate aldolase (HOGA1; PH3), resulting in increased hepatic oxalate production [1,7]. Secondary hyperoxaluria includes enteric hyperoxaluria, which occurs in association with fat malabsorption following bariatric surgery, inflammatory bowel disease, or short-bowel syndromes, as well as dietary hyperoxaluria and conditions associated with reduced intestinal oxalate degradation by commensal microbiota [8,9,10]. In individuals without a monogenic disorder, dietary factors represent an important modifiable determinant of oxalate burden. The bioavailability of dietary oxalate depends not only on total oxalate intake, but also on interactions with the gut bacteriome, dietary calcium, magnesium, and fibre. Consequently, diets low in calcium may substantially increase the intestinally absorbable fraction of oxalate [11]. In primary hyperoxaluria, genotype strongly influences disease severity and prognosis; for example, age at kidney failure in PH1 varies substantially among different *AGXT* variants, highlighting the genetic heterogeneity that must be considered when interpreting findings from experimental models [7,12].

Advances in understanding hyperoxaluria pathogenesis and the development of emerging therapies have relied heavily on in vivo models. Animal systems enable the controlled induction of hyperoxaluria, reproducible assessment of calcium oxalate crystal deposition, and systematic evaluation of dietary, pharmacological, microbial, and genetic interventions within the context of an intact organism, taking into account renal architecture, immune responses, and the gut–kidney axis—features that cannot be fully reproduced in vitro [13,14,15,16]. However, experimental models vary considerably in the aspects of human disease that they reproduce, and each model has specific advantages, limitations, and potential confounders. This review focuses exclusively on in vivo models of hyperoxaluria. Firstly, the criteria for evaluating model validity and relevant experimental endpoints are discussed, followed by an overview of chemically and dietary induced models, genetic models, enteric and microbial models, and large-animal (porcine) models. Finally, the way in which these systems have contributed to the development of current and emerging therapeutic strategies, with particular emphasis on oxalate decarboxylase-based and other gut-directed approaches is examined. The aim of the current review was to provide a critical framework for selecting, interpreting, and integrating in vivo models in hyperoxaluria research.

## 2. Literature Search Strategy

This narrative review was based on a structured, non-systematic literature search and was not intended to constitute a formal systematic review or meta-analysis. Literature was identified through searches in PubMed, Web of Science, Scopus, and Google Scholar, covering publications from database inception to July 2026. Representative search terms included those related to hyperoxaluria and oxalate-associated kidney disease (“hyperoxaluria”, “primary hyperoxaluria”, “enteric hyperoxaluria”, “calcium oxalate”, “nephrolithiasis”, “urolithiasis”, “oxalate nephropathy”, “nephrocalcinosis”), experimental models and biological mechanisms (“animal model”, “in vivo”, “mouse”, “rat”, “knockout”, “Agxt”, “Grhpr”, “Hoga1”, “Slc26a6”, “osteopontin”, “Tamm–Horsfall protein”, “Drosophila”, “pig”, “porcine”), and therapeutic strategies (“RNA interference”, “lumasiran”, “nedosiran”, “gene editing”, “CRISPR”, “base editing”, “stiripentol”, “oxalate decarboxylase”, “reloxaliase”, “*Oxalobacter formigenes*”, “probiotic”, and “faecal microbiota transplantation”).

Priority was given to peer-reviewed original research articles describing the development, characterisation, or application of in vivo models of hyperoxaluria and calcium oxalate-associated disease. Relevant review articles were additionally consulted to provide historical and mechanistic context and to identify seminal publications. Reference lists of retrieved articles were screened manually, and only English-language publications were considered. The literature was synthesized narratively, with particular emphasis on the mechanism of model induction, the principal phenotype reproduced, experimental endpoints, and the construct, face, and predictive validity of individual models. Publications that did not involve in vivo experimental models were generally considered only when necessary to provide mechanistic or translational context.

## 3. Validity Criteria and Endpoints for In Vivo Models of Hyperoxaluria

**Figure 1:**
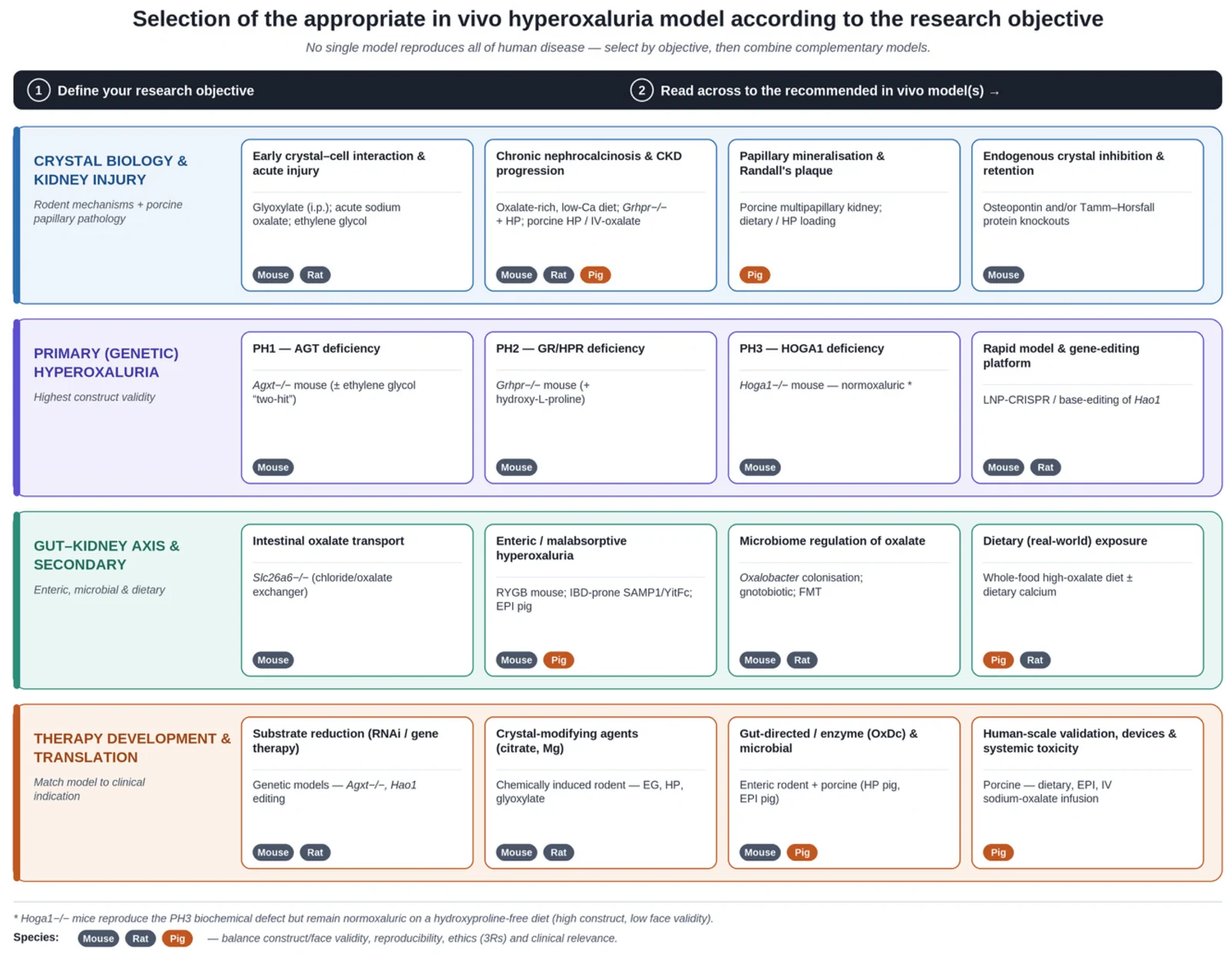
Framework for selecting appropriate in vivo hyperoxaluria models according to the research objective. The framework links major research objectives with representative animal models and species, highlighting their complementary roles in studying crystal biology and renal injury, primary and secondary hyperoxaluria, the gut–kidney axis, and therapeutic development. Model selection should be guided by the specific biological question and the balance between construct and face validity, reproducibility, ethical considerations, and translational relevance. Abbreviations: CKD, chronic kidney disease; EPI, exocrine pancreatic insufficiency; FMT, fecal microbiota transplantation; HP, hydroxy-L-proline; IBD, inflammatory bowel disease; LNP, lipid nanoparticle; OxDc, oxalate decarboxylase; PH1–PH3, primary hyperoxaluria types 1–3; RYGB, Roux-en-Y gastric bypass.

A useful model of hyperoxaluria should be evaluated across three complementary dimensions of validity (Figure 1). Construct validity refers to whether the model reproduces the causal mechanism underlying the human condition—for example, hepatic oxalate overproduction in primary hyperoxaluria (PH) or intestinal oxalate hyperabsorption in malabsorptive states. Face validity reflects the extent to which the resulting phenotype resembles human disease, including biochemical features of hyperoxaluria, patterns of crystal formation, tissue injury, and disease progression. Predictive validity refers to whether interventions that modify the model produce therapeutic effects that translate to patients, representing the ultimate rationale for preclinical model development [14,16]. These dimensions do not necessarily coincide: a model may accurately reproduce a molecular mechanism while generating an atypical phenotype or may induce convincing crystalluria through a pathway with limited relevance to human disease.

A central prerequisite for meaningful comparison between experimental systems is the distinction between related but non-interchangeable phenotypes. Hyperoxaluria refers specifically to increased urinary oxalate excretion and does not necessarily imply crystal formation or renal injury. Crystalluria describes the presence of crystals in the urine and is associated with urinary supersaturation and crystallization but does not establish tissue retention or stone formation. Nephrocalcinosis refers to calcium deposition within the renal parenchyma and may involve intratubular, interstitial, and papillary compartments. Oxalate nephropathy denotes renal injury associated with oxalate crystal deposition and may involve tubular damage, inflammation, interstitial fibrosis, and progressive loss of kidney function. Finally, nephrolithiasis refers to the formation of discrete, macroscopic calculi within the urinary collecting system. In humans, calcium oxalate stones typically develop over prolonged periods through mechanisms including overgrowth of Randall’s plaques, plugging of the ducts of Bellini, or free-solution crystallization. Thus, nephrolithiasis should not be inferred solely from crystalluria or intrarenal crystal deposition. These phenotypes represent different stages or consequences of disturbed oxalate homeostasis and may coexist, but they should not be used interchangeably. Importantly, many chemically induced rodent models reproduce hyperoxaluria, crystalluria, and intratubular calcium oxalate deposition without reproducing the chronic papillary pathology or macroscopic stone formation characteristic of human calcium oxalate nephrolithiasis. Consequently, the phenotype reproduced by each model should be explicitly defined when evaluating its face validity and translational relevance.

A further recurring limitation of numerous experimental hyperoxaluria models is the mismatch in disease timescale. Most chemically induced rodent models rely on acute or subacute increases in urinary oxalate and reproduce early events such as crystal nucleation, aggregation, tubular retention, and acute injury over days to weeks. However, they do not recapitulate the prolonged natural history of chronic human calcium oxalate stone disease, including the progressive development of Randall’s plaque, papillary mineralisation, or ductal plugging [15,17,18]. In humans, CaOx stones arise through several pathways, including overgrowth on exposed interstitial apatite deposits (Randall’s plaque), plugging of the ducts of Bellini, and free-solution crystallization. These mechanisms differ in their relevance to experimental models and influence how faithfully a given system represents human disease [15,17,18]. Therefore, experimental models should be selected according to the biological question being addressed—such as acute crystal–cell interactions, chronic nephrocalcinosis and CKD progression, intestinal oxalate handling, or genetically determined oxalate overproduction—rather than according to the expectation that a single model can reproduce all aspects of hyperoxaluria and the associated renal consequences.

Meaningful comparison between studies requires consistent and standardised outcome measures. Core biochemical endpoints include 24-h urinary oxalate excretion, which is generally normalized to urinary creatinine when measured in spot urine samples, urinary glycolate and, in PH3 models, hydroxyoxoglutarate-related metabolites. In advanced disease states, plasma oxalate concentrations may provide an additional indicator of systemic oxalate burden. Crystal-related endpoints include crystalluria assessed by polarised-light microscopy, allowing differentiation between CaOx monohydrate and dihydrate crystals, and quantitative assessment of tissue crystal deposition. For the latter, Pizzolato and Yasue metal-substitution histochemical techniques provide greater specificity for CaOx identification than routine haematoxylin–eosin staining alone. Renal outcomes include histopathological assessment of tubular injury and fibrosis, markers of inflammation and oxidative stress, and functional parameters such as creatinine clearance, cystatin C, and blood urea nitrogen. Consistent reporting of experimental variables—including the induction method and dose, duration of exposure, dietary composition (particularly calcium, oxalate, magnesium, and vitamin content), animal strain, sex, and age—is essential because each may substantially influence disease expression and treatment response. Table 1 summarises the major in vivo systems according to these validity criteria.

## 4. Acquired Hyperoxaluria Models: Chemical, Dietary, and Metabolic Approaches

### 4.1. Ethylene Glycol

The ethylene glycol (EG)-induced rat model is one of the most widely used and best-characterised experimental systems for the study of CaOx nephrolithiasis. EG is typically administered at concentrations of 0.5–1.0% in drinking water and undergoes hepatic metabolism through glycolaldehyde, glycolate, and glyoxylate to oxalate, resulting in a robust and reproducible hyperoxaluric state. This is accompanied by intratubular CaOx deposition, tubular dilation, epithelial injury, oxidative stress, and inflammatory responses within days to weeks [16,19,20]. Ammonium chloride is frequently co-administered to induce mild metabolic acidosis and reduce urinary pH, thereby facilitating calcium oxalate crystallization. Much of the current understanding of intratubular crystal retention, including the “fixed-particle” mechanism whereby crystals adhere to injured tubular epithelium and form a nidus rather than being eliminated with urine, has been derived from EG-based and acute oxalate-salt models [16,21].

The major advantages of the EG model are its simplicity, reproducibility, relatively low cost, and suitability for screening potential antiurolithic interventions. However, its interpretation is limited by the fact that EG and several metabolic intermediates can exert direct nephrotoxic effects. Moreover, EG toxicity affects other organs (especially the CNS and liver) and there is a risk of developing metabolic acidosis, which limits its use [21]. At higher doses or with prolonged exposure, tubular necrosis and systemic toxicity may develop, complicating the distinction between oxalate-mediated injury and nonspecific toxic effects [20,21]. Furthermore, the commonly used 0.75% EG concentration does not consistently reproduce the same metabolic disturbances across studies, and experimental outcomes are strongly influenced by animal strain and sex [22]. An additional limitation of EG-based models is related to the mechanism by which hyperoxaluria is induced. Although oral administration partially resembles the natural route of exposure, EG acts primarily as a metabolic precursor of oxalate rather than as a source of intestinal oxalate. Following hepatic conversion to glycolate, glyoxylate, and oxalate, it increases endogenous oxalate production rather than reproducing the complex interaction between dietary oxalate intake, intestinal absorption, microbial degradation, and renal excretion observed in humans. Therefore, EG-based models have limited applicability for investigating the contribution of the intestinal microbiome to oxalate homeostasis [23]. Administration of EG may itself alter the intestinal environment and microbial composition, creating a chemically modified system in which microbiome changes may reflect compound-specific effects rather than oxalate-driven mechanisms. This represents an important translational limitation when evaluating therapies targeting intestinal oxalate degradation, including oxalate-degrading enzymes.

### 4.2. Hydroxy-L-Proline

Hydroxy-L-proline (HP), a naturally occurring amino acid derivative generated during collagen turnover, is metabolised to glyoxylate and subsequently to oxalate, thereby increasing endogenous oxalate production without administration of a synthetic xenobiotic [20,24,25]. Dietary HP supplementation, commonly at concentrations of 3–10%, produces dose-dependent hyperoxaluria and calcium oxalate crystal formation. Optimised protocols can generate sustained hyperoxaluria with comparatively limited direct nephrotoxicity, making the HP model particularly valuable for studies of crystal–epithelial interactions and longer-term consequences of elevated oxalate exposure [20,24,26].

The principal limitation of this model is that HP metabolism generates several intermediates, including glyoxylate-related metabolites, and renal effects cannot always be attributed exclusively to oxalate itself. Similar to EG, HP induces hyperoxaluria through hepatic metabolic conversion to glyoxylate and subsequently oxalate, rather than through increased intestinal oxalate exposure. Therefore, although HP represents a more physiological endogenous precursor than synthetic compounds, it does not fully reproduce dietary oxalate-driven hyperoxaluria or the contribution of intestinal oxalate handling and microbiota-mediated oxalate degradation to disease development [23].

### 4.3. Soluble Oxalate Salts

Direct administration of soluble oxalate salts provides a method to increase oxalate exposure while bypassing endogenous hepatic oxalate synthesis pathways, allowing more precise control of the oxalate stimulus. Acute administration of sodium oxalate, particularly by intraperitoneal injection, produces a rapid increase in urinary oxalate excretion and almost immediate formation of calcium oxalate crystals within proximal tubular lumina. Subsequent crystal deposition occurs predominantly at the corticomedullary junction and papillary regions, with crystal number, size, and distribution varying according to dose and exposure conditions. These models have been instrumental in defining early mechanisms of tubular crystal adhesion, retention, and injury [16,27].

However, injection-based administration of soluble oxalate salts, particularly intraperitoneal sodium oxalate, bypasses the gastrointestinal tract and therefore does not account for intestinal oxalate absorption, dietary oxalate handling, or microbiota-mediated oxalate degradation. Moreover, these models typically represent acute oxalate overload rather than chronic exposure, with crystal formation driven by rapid increases in systemic oxalate concentrations. Consequently, although highly informative for investigating early crystal–epithelial interactions and mechanisms of acute renal injury, they have limited applicability for modelling the chronic and multifactorial pathogenesis of human calcium oxalate nephrolithiasis [23].

More chronic dietary oxalate models extend this approach toward clinically relevant oxalate nephropathy. Dietary oxalate supplementation increases urinary oxalate in a controlled manner and avoids some confounding effects associated with EG and HP metabolism, making these approaches particularly useful for genetic and mechanistic studies of susceptibility to oxalate-induced renal injury. In mice, soluble oxalate-rich and calcium-deficient diets can induce progressive nephrocalcinosis accompanied by tubular injury, interstitial inflammation, fibrosis, and systemic manifestations of CKD, including anaemia, metabolic acidosis, hyperphosphatemia, fibroblast growth factor 23 (FGF23) activation, hypertension, and cardiac fibrosis [28,29]. Activation of the NLRP3 inflammasome and IL-1β-dependent inflammatory signaling represents a key mechanism linking crystal deposition to renal inflammation and disease progression, as Nlrp3-deficient animals show reduced injury and protection from CKD development [28,29]. Similar oxalate-diet approaches in Dahl salt-sensitive rats have further extended these observations to models incorporating hypertensive kidney disease [30]. Together, these systems provide a continuum between acute crystal-induced injury and chronic oxalate-associated nephropathy.

### 4.4. Glyoxylate-Induced Hyperoxaluria and Experimental Modifiers of Oxalate Injury

Direct glyoxylate administration, typically through repeated intraperitoneal injections in mice, represents a widely used model of acute calcium oxalate crystal nephropathy. Glyoxylate is rapidly converted to oxalate, increasing endogenous oxalate production and inducing tubular mitochondrial injury and oxidative stress within hours, followed by intratubular CaOx crystal formation within several days. This makes the model particularly suitable for investigating early crystal–cell interactions, oxidative pathways, and mechanisms of crystal-induced inflammation [31,32]. Glyoxylate-based models have contributed substantially to defining the sequence linking tubular epithelial injury, reactive oxygen species generation, crystal nucleation, and retention, and have been used to evaluate antioxidant and anti-inflammatory strategies [28,31].

Nevertheless, the common use of intraperitoneal glyoxylate administration represents an important translational limitation. As with other parenteral oxalate-generating approaches, the model is not suitable for investigating intestinal mechanisms of oxalate handling, or microbiota-mediated oxalate degradation. Furthermore, repeated administration of high glyoxylate doses may preferentially model acute crystal-induced injury rather than the chronic and multifactorial development of calcium oxalate nephrolithiasis [23].

In addition to the primary oxalate-generating stimulus, several dietary interventions are commonly used to modify disease severity. Magnesium deficiency accelerates crystalluria and CaOx deposition, whereas magnesium supplementation can attenuate these effects. Vitamin B6 (pyridoxine) deficiency impairs glyoxylate metabolism and increases oxalate production, while dietary calcium availability directly influences intestinal oxalate absorption and urinary oxalate excretion [11,24]. These modifiers provide valuable experimental tools for dissecting mechanisms of susceptibility; however, they also introduce additional variables that may reduce similarity to spontaneous human disease and should therefore be reported explicitly.

### 4.5. Species, Strain, and Sex as Determinants of Experimental Outcome

The phenotype generated by an experimental hyperoxaluria protocol is strongly influenced by the genetic and physiological background of the animal. Consequently, the induction method alone does not define the model, and species, strain, sex, age, and renal maturity should be considered when comparing results across studies.

Strain-dependent differences are particularly evident in chemically induced rodent models. For example, Wistar rats accumulate more renal CaOx after EG administration than Fischer 344 rats, reflecting higher plasma oxalate, greater urinary CaOx supersaturation, and variation in urinary macromolecules and protein composition that influence crystal nucleation and retention [33,34]. The distinction is not attributable to oxalate load alone: modification or removal of the urinary protein fraction narrows the difference between the two strains, indicating that strain-specific inhibitor capacity determines whether a given degree of supersaturation proceeds to crystal retention [33,34]. Comparable divergence occurs in mice, in which susceptibility to glyoxylate- and oxalate-induced renal CaOx deposition differs appreciably between commonly used mice with inbred backgrounds. This has direct consequences for genetic models, because a substantial proportion of *Agxt*-deficient males with susceptible backgrounds develop urolithiasis, but show a much milder phenotype compared to others, so the same targeted allele can yield different crystal burdens depending on the background strain and the degree of backcrossing. Strain should therefore be treated as an experimental variable, and comparisons between studies performed on strains with different backgrounds should be made with caution.

Sex also substantially modifies disease expression. Experimental evidence indicates that androgens promote, whereas oestrogens attenuate CaOx crystal deposition. Consequently, intact male rodents generally develop more extensive crystal deposition and renal injury than females or castrated males under comparable hyperoxaluric conditions [35]. Studies should therefore report sex explicitly and avoid generalizing results obtained in one sex to both sexes without supporting evidence.

Age represents an additional source of variation. Renal function, glomerular filtration rate, urinary composition, and oxalate handling change during development. This issue is particularly relevant in rapidly growing animals, in which changes in renal maturation may complicate longitudinal interpretation of urinary oxalate excretion. Where appropriate, oxalate excretion should therefore be interpreted using appropriate normalization, such as the urinary oxalate-to-creatinine ratio, together with markers of renal function.

These biological variables should not be considered merely as methodological details. They may influence whether a given experimental protocol produces isolated hyperoxaluria, crystalluria, nephrocalcinosis, or clinically relevant renal injury. Their systematic reporting and incorporation into comparative analyses are therefore essential for improving reproducibility and interpretation across experimental systems [22].

## 5. Genetic Models of Primary Hyperoxaluria

Genetically engineered mouse models provide the highest level of construct validity for the primary hyperoxalurias because they reproduce the underlying enzymatic defects responsible for endogenous hepatic oxalate overproduction [7,36,37]. Consequently, these models have become indispensable for investigating disease mechanisms and for the preclinical evaluation of substrate-reduction, gene replacement, and gene-editing therapies. Complementary knockout models targeting intestinal oxalate transporters and endogenous inhibitors of crystal formation further dissect the pathways governing oxalate homeostasis, crystal retention, and renal injury after oxalate production has occurred.

### 5.1. PH1: The Agxt-Deficient Mouse

Targeted disruption of *Agxt* produces mice that develop normally but exhibit elevated plasma and urinary glycolate, increased urinary oxalate excretion, crystalluria, and, on susceptible genetic backgrounds, calcium oxalate urolithiasis in a substantial proportion of male animals [38]. Although spontaneous nephrocalcinosis is generally mild and develops gradually with age, additional oxalate loading, most commonly using EG, induces severe nephrocalcinosis, progressive renal injury, and renal dysfunction, creating a flexible “two-hit” model of disease progression [38].

The *Agxt*-deficient mouse has served as the principal preclinical platform for therapeutic development in PH1. Restoration of hepatic AGT expression by adenoviral and adeno-associated viral (AAV) vectors corrects the biochemical phenotype [38,39], while the same model has provided proof of concept for RNA interference-based substrate reduction and oral oxalate decarboxylase therapy. Its principal limitation is the relatively mild spontaneous renal phenotype, meaning that additional oxalate challenge is generally required to reproduce progressive nephrocalcinosis and kidney injury.

### 5.2. PH2: The Grhpr-Deficient Mouse

*Grhpr*-knockout mice reproduce the biochemical abnormalities of PH2 and develop marked hyperoxaluria with calcium oxalate crystal formation. Following an HP challenge, these animals exhibit a severe and highly reproducible phenotype characterised by extensive nephrocalcinosis, progressive deterioration of renal function, and increasing plasma cystatin C concentrations over several weeks [40]. Compared with *Agxt*-deficient mice, disease progression is more consistent and substantially more severe.

The model has proven particularly valuable for therapeutic evaluation. Pharmacological inhibition of hydroxyproline dehydrogenase (HYPDH) using N-propargylglycine markedly improves survival while preventing stone formation, tubular injury, and renal dysfunction [41,42]. However, as in PH1, accelerated disease progression depends largely on dietary HP loading, representing an important limitation when modelling spontaneous human disease.

### 5.3. PH3: The Hoga1-Deficient Mouse

*Hoga1*-knockout mice reproduce the characteristic biochemical alterations of PH3, including elevated plasma and urinary concentrations of 4-hydroxy-2-oxoglutarate and 2,4-dihydroxyglutarate together with altered hydroxyproline metabolism [43]. In contrast to patients with PH3, however, these animals fail to develop spontaneous hyperoxaluria when maintained on a hydroxyproline-free diet. This discrepancy is thought to result from species-specific differences in glyoxylate metabolism, particularly the persistence of mitochondrial AGT activity in mice, whereas human AGT is confined to peroxisomes.

Accordingly, the current PH3 mouse model exhibits excellent construct validity but limited face validity. This illustrates an important principle of translational modelling: faithful reproduction of the causal mutation does not necessarily guarantee reproduction of the clinical phenotype, particularly when metabolic pathways differ between species. Consequently, the utility of the existing PH3 model for therapeutic efficacy studies remains limited [43].

### 5.4. Rapidly Engineered and Human-Relevant Genetic Models

Recent advances in somatic genome engineering have substantially accelerated the development of experimental hyperoxaluria models. Lipid nanoparticle (LNP)-mediated delivery of CRISPR–Cas9 has been used both to generate PH1 models and to evaluate therapeutic genome editing within the same experimental platform. A single intravenous administration of LNP-delivered CRISPR–Cas9 targeting hepatic *Hao1* (glycolate oxidase) produced sustained therapeutic effects throughout a 12-month follow-up period with no significant detectable off-target effects, liver toxicity, or substantial immune response, while LNP-mediated base editing of *Hao1* achieved durable metabolic correction in PH1 rats, with normalization of urinary oxalate maintained for at least 6 months [44,45].

Two safety questions dominate the translation of these approaches and are an important reason why preclinical animal models remain necessary. The first is editing specificity. Nuclease-based editing acts through double-strand breaks and can generate off-target insertions and deletions, large on-target deletions, chromosomal rearrangements, and p53-dependent responses in edited hepatocytes. Base editors avoid double-strand breaks but introduce distinct liabilities, including bystander editing within the editing window and guide-independent deamination of DNA or RNA. Off-target editing was low in the *Hao1*-targeted studies cited above, but hepatocyte editing is permanent and cannot be withdrawn if a late adverse effect emerges. Genome-wide unbiased off-target assessment and long-term follow-up therefore remain essential [44,45]. A consideration specific to *HAO1* is that the strategy deliberately creates a state resembling glycolate oxidase deficiency; although lifelong loss of *HAO1* function appears to be well tolerated in the limited human evidence available, the consequences of complete enzymatic ablation across the lifespan remain incompletely established [44]. The second question is delivery. Systemically administered LNPs show strong hepatic tropism and have clinical precedent, but their use may be associated with infusion-related reactions, complement activation, and transient transaminase elevations. Their transient exposure may also necessitate redosing, for which anti-PEG immunity represents a potential constraint. AAV vectors provide durable expression but are limited by pre-existing and treatment-induced neutralizing antibodies, dose-dependent hepatotoxicity, and potential long-term safety concerns associated with persistent vector genomes. Preclinical studies reporting metabolic correction should therefore assess not only therapeutic efficacy but also off-target editing, liver histopathology, immunogenicity, and durability. These parameters are critical for determining the clinical feasibility of genome-editing approaches for hyperoxaluria.

These approaches markedly shorten the time required for model generation and therapeutic validation, thereby accelerating preclinical development of gene-editing strategies currently progressing toward clinical application. Nevertheless, somatic genome editing cannot fully reproduce the developmental and lifelong consequences of germline enzyme deficiency. Germline knockout models therefore remain important reference systems for studies of disease pathogenesis and natural history.

### 5.5. Transporter and Crystal-Retention Knockout Models

A complementary group of genetically modified models has been used to investigate oxalate handling rather than oxalate production. Mice lacking the apical chloride/oxalate exchanger Slc26a6 developed enteric hyperoxaluria, hyperoxalemia, and a high incidence of calcium oxalate urolithiasis because impaired intestinal oxalate secretion increases net oxalate absorption. Restriction of dietary oxalate markedly attenuates this phenotype, confirming the central role of intestinal transport in systemic oxalate homeostasis [46,47].

Beyond intestinal absorption, Slc26a6 also contributes to protective enteric oxalate secretion during declining renal function, thereby linking intestinal transport to systemic oxalate balance in CKD. The identification of a dominant-negative *SLC26A6* variant causing inherited enteric hyperoxaluria in humans provides an uncommon example of direct concordance between mouse genetics and human disease [48,49]. These models have therefore established the intestine as an active and therapeutically accessible regulator of oxalate homeostasis, providing a rationale for transporter-targeted and gut-directed interventions.

A second group of knockout models focused on endogenous inhibitors of crystal formation. Mice lacking osteopontin or Tamm–Horsfall protein individually exhibit only limited spontaneous renal calcification; however, a combined deficiency of both resulted in extensive intratubular calcium oxalate crystallization and stone formation following an hyperoxaluric challenge, whereas single knockouts remained largely protected [50,51]. These findings demonstrate that urinary macromolecules function as partially redundant physiological inhibitors of crystal nucleation, aggregation, and retention. Collectively, transporter and crystal-retention models complement the hepatic overproduction models described above by dissecting the sequential biological processes that determine whether an oxalate load ultimately progresses to clinically significant crystal deposition and kidney injury.

## 6. Secondary and Enteric Hyperoxaluria—Modelling the Gut–Kidney Axis

Enteric hyperoxaluria—defined clinically as a urinary oxalate above ~40 mg/24 h in the setting of gastrointestinal disease with fat malabsorption—has historically been under-modelled relative to its clinical importance, but recent systems now capture its mechanism [9,10,52]. Untreated, it is a recognised cause of oxalate nephropathy and irreversible renal failure, most notably after Roux-en-Y gastric bypass [53].

### 6.1. Malabsorptive and Inflammatory Bowel Models

Two complementary murine models have been able to reproduce the principal clinical drivers of enteric hyperoxaluria. In the surgical model, diet-induced obese mice undergo Roux-en-Y gastric bypass and are then maintained on a high-fat, oxalate-enriched diet, resulting in the development of fat malabsorption and hyperoxaluria that mirror post-bariatric enteric hyperoxaluria. In the inflammatory model, ileitis-prone SAMP1/YitFc mice on high-fat, oxalate-supplemented diets develop malabsorptive hyperoxaluria analogous to Crohn’s disease [54]. Both reproduce increased stool fat and elevated urinary oxalate and allow for the investigation of how bile acids, luminal free fatty acids, and mucosal permeability promote oxalate absorption. These models are technically demanding and phenotypically variable but provide the construct validity that dietary loading alone cannot.

### 6.2. Gnotobiotic, Human Microbiota-Associated, and Faecal Transplantation Models

Germ-free and gnotobiotic animals, selective colonisation, and faecal microbiota transplantation (FMT) have established that the intestinal microbiome is a bona fide regulator of oxalate homeostasis rather than a passive bystander [13]. Clinical and translational studies reinforce this, and stone formers have been shown to carry a distinct faecal microbiome with reduced oxalate-degrading functional capacity relative to non-stone formers, with the gut–kidney axis in nephrolithiasis extending well beyond *Oxalobacter* alone [55,56,57]. Transplantation of faecal microbiota into hyperoxaluric rats significantly reduced CaOx stone formation and renal injury by restoring beneficial microbiota taxa and intestinal barrier function, demonstrating a causal and transferable microbial effect [58]. Such models are indispensable for dissecting which microbial functions—oxalate degradation, barrier maintenance, or bile-acid handling—mediate protection, and they provide the rationale for microbial and enzymatic therapies. Conversely, bacterial biofilms can provide a scaffold for crystal nucleation and aggregation, a reminder that the microbiome’s influence on stone disease is bidirectional [59,60].

### 6.3. Oxalobacter formigenes Colonization Models

*Oxalobacter formigenes*, an obligate anaerobe that uses oxalate as its sole carbon and energy source, occupies a special place amongst the bacterial colonisation models. Its absence from the intestinal tract is associated with CaOx stone disease in humans [61,62], and in rodents *Oxalobacter formigenes* colonisation lowers urinary oxalate through two mechanisms: intraluminal oxalate degradation and, distinctively, the induction of net enteric (colonic) oxalate secretion that reduces the systemic oxalate burden [63]. This secretory effect does not require the apical transporters Slc26a3 or Slc26a6, indicating additional pathways of microbe–host oxalate exchange. In a gastric-bypass rat model, *Oxalobacter* colonisation reduced urinary oxalate by up to ~74% [64]. These findings make the organism both a mechanistic probe of the gut–kidney axis—now a well-developed conceptual framework linking the microbiome, host oxalate transporters, and renal function—and a candidate therapeutic [13,65].

## 7. Dietary Hyperoxaluria and Models of Real-World Oxalate Exposure

Dietary hyperoxaluria occupies a distinct and clinically important position between the primary and enteric forms of hyperoxaluria, and it is the variant most relevant to the large population of idiopathic calcium stone formers. In people with normal glyoxylate metabolism and intact intestinal function, approximately half of urinary oxalate is derived from the diet and half from endogenous synthesis, making diet the single largest modifiable determinant of urinary oxalate [66,67]. Crucially, that contribution is not fixed: the fraction of ingested oxalate reaching the urine varies several-fold with the composition of the meal in which it is eaten. This is precisely why a dietary model cannot be reduced to a dose of soluble oxalate salt.

Three factors dominate oxalate bioavailability. First, chemical form: soluble oxalate, as in raw spinach or rhubarb, is far more bioavailable than the insoluble calcium-bound oxalate that predominates in foods such as black beans, so total oxalate content is a poor proxy for absorbable oxalate. Second, co-ingested calcium is the principal modulator—Ca^2+^ binds oxalate in the intestinal lumen to form insoluble complexes excreted in faeces, and reducing calcium intake from roughly 1000 to 400 mg/day raises the proportion of ingested oxalate appearing in urine from between 20–40% to approximately 50% [66,67]. Magnesium, phytate and dietary fiber act similarly, but seem to have a less powerful effect to that of Ca^2+^. Third, dietary oxalate precursors contribute to any measurement of food oxalate on its own: including hydroxyproline from collagen, glycolate, and ascorbate (vitamin C), which degrade non-enzymatically to oxalate [67]. This explains why identical oxalate intakes can produce markedly different urinary responses between individuals and between experimental diets.

That these interactions are physiologically consequential has been shown directly in humans: a single oxalate-rich meal raises urinary oxalate within hours and induces a measurable increase in urinary calcium oxalate nanocrystals, demonstrating that ordinary dietary exposure—not merely extreme loads—can transiently drive urine into a crystallizing state [68]. Chronic, low-grade dietary oxalate exposure is increasingly implicated in kidney function decline beyond stone formation, and in the systemic consequences of oxalate accumulation [69].

This has direct implications for the design of hyperoxaluria models. Administering soluble potassium or sodium oxalate delivers a defined, highly reproducible load, but strips away the food matrix, the calcium–oxalate interaction, and the digestive processing that determine real human exposure; such models therefore combine high internal validity with low dietary realism. Whole-food, “humanised” high-oxalate diets—built from foods such as rhubarb, spinach, tomato juice and potato—restore that realism at the cost of reproducibility, because the oxalate content of plant material varies with cultivar, season, soil and preparation, and boiling can roughly halve the soluble oxalate of rhubarb. Studies using such diets should therefore measure, rather than estimate, the soluble oxalate content of the final feed, and should report dietary calcium explicitly, since the calcium-to-oxalate ratio may influence urinary oxalate as much as the oxalate dose itself.

### Naturally Occurring Animal Models of Calcium Oxalate Stone Disease

Dogs and cats develop CaOx urolithiasis spontaneously and therefore provide a conceptually distinct form of translational evidence compared with experimentally induced models. Their disease arises in outbred populations exposed to heterogeneous genetic, dietary, environmental, and metabolic influences and may recur over time, providing an opportunity to investigate stone formation under naturally occurring conditions. CaOx has remained one of the predominant urolith types in both species, although its relative frequency has changed over time and differs among populations. Earlier epidemiological studies documented an increase in the proportion of CaOx-containing uroliths accompanied by a decline in struvite-containing uroliths, whereas more recent large-scale analyses from North American urolith databases reported a subsequent decline in the proportion of CaOx-containing uroliths in both dogs and cats. Thus, temporal trends in urolith composition are not uniform and may reflect changes in diet, management, breed distribution, and other environmental factors rather than a single causal mechanism [70,71,72].

Several risk factors for CaOx urolithiasis in dogs and cats overlap with those recognised in humans, although their relative importance differs between species. In dogs, increasing age, male sex, neutering status, overweight or obesity, breed, and abnormalities that increase urinary calcium excretion are associated with CaOx urolithiasis. Well-described breed predispositions include Miniature Schnauzers, Lhasa Apsos, Yorkshire Terriers, Bichon Frisés, Shih Tzus, and Miniature Poodles. In cats, increased risk has been reported in several breeds, including Persian, Himalayan, Burmese, British Shorthair, Ragdoll, and other breed groups, although the magnitude and consistency of these associations vary among populations. These breed-related differences support a genetic contribution to susceptibility and provide an opportunity to investigate interactions between genetic background and environmental or metabolic factors [70,73,74].

Metabolic and urinary factors are particularly relevant to CaOx formation in companion animals. Hypercalciuria is an important risk factor in dogs and cats, while hypercalcemia may increase urinary calcium excretion and predispose to CaOx urolith formation. In cats with naturally occurring CaOx urolithiasis, hypercalcemia, including idiopathic hypercalcemia, has been reported relatively frequently. In dogs, disorders associated with increased urinary calcium excretion, including hypercalcemia and hyperadrenocorticism, are recognised risk factors for CaOx urolithiasis. Dietary composition may additionally influence urinary calcium and oxalate concentrations, urine volume, and calcium oxalate supersaturation. However, although dietary oxalate can increase urinary oxalate availability, urinary oxalate appears to play a less prominent role in naturally occurring CaOx urolithiasis in cats and dogs than in humans, whereas urinary calcium appears to be relatively more important [70,75].

Importantly, spontaneous CaOx urolithiasis in companion animals should not be equated with hyperoxaluria. Most naturally occurring CaOx urolithiasis in adult cats and dogs is not characterised by the marked hyperoxaluria typical of primary hyperoxaluria, and CaOx stone formation may occur in the context of relatively modest urinary oxalate excretion. Companion animals may therefore be particularly informative for investigating idiopathic CaOx stone formation and the interaction between urinary calcium, oxalate, inhibitors of crystallization, diet, and other metabolic factors rather than serving primarily as models of systemic oxalate overproduction [70]. An important exception is the naturally occurring form of primary hyperoxaluria described in cats. Affected related cats were reported to develop hyperoxaluria and renal CaOx deposition, and the disorder was subsequently shown to result from a mutation in the feline GRHPR gene. This condition corresponds to primary hyperoxaluria type 2 (PH2), which in humans is caused by deficiency of glyoxylate reductase/hydroxypyruvate reductase. The feline GRHPR mutation therefore represents a naturally occurring genetic model relevant specifically to PH2 rather than to primary hyperoxaluria as a whole [70,76].

The extent to which common canine and feline CaOx urolithiasis reproduces the renal pathology of human idiopathic CaOx nephrolithiasis remains incompletely characterised. In humans, idiopathic CaOx nephrolithiasis is closely associated with Randall’s plaques and, in some patients, ductal plugging. Dogs and cats can develop renal mineralisation and CaOx uroliths, and recurrent disease has been documented, but the occurrence, distribution, temporal development, and pathological significance of lesions analogous to the human pathways of papillary stone formation have not been characterised sufficiently to establish full mechanistic equivalence. The available comparative literature therefore identifies companion animals as promising naturally occurring systems while also emphasising substantial knowledge gaps in the renal pathogenesis of their disease [70].

The principal strength of companion animals as naturally occurring models is their ability to capture spontaneous stone formation, disease heterogeneity, recurrence, and interactions between genetic susceptibility and long-term environmental and metabolic influences. Their relatively shorter lifespan compared with humans may also permit investigation of recurrence and disease progression over a shorter experimental timeframe. At the same time, naturally occurring disease is inherently less standardised than experimentally induced disease. Affected animals are client-owned, dietary and environmental exposures cannot be fully controlled, longitudinal sampling is often limited, and renal tissue is generally available only under selected clinical circumstances. These characteristics constrain mechanistic experimentation and make direct comparison between studies more difficult. Companion-animal CaOx urolithiasis should therefore be viewed as complementary to, rather than interchangeable with, controlled experimental models. Rodent and genetically engineered models remain better suited to mechanistic studies of oxalate metabolism, defined genetic defects, and targeted therapeutic interventions, whereas spontaneous disease in dogs and cats provides an opportunity to investigate idiopathic stone formation, genetic susceptibility, recurrence, dietary and environmental influences, and longer-term disease progression under naturally occurring conditions. The naturally occurring feline GRHPR-associated PH2 phenotype additionally provides a distinct genetic model for studying primary hyperoxaluria type 2. Overall, the greatest translational value of companion animals lies in complementing highly controlled experimental systems with naturally occurring disease in genetically heterogeneous animals exposed to clinically relevant environmental and metabolic influences.

## 8. Large-Animal and Translational Porcine Models

### 8.1. Rationale for the Pig Model

The domestic pig (*Sus scrofa domestica*) represents a complementary large-animal model for investigating selected aspects of hyperoxaluria and CaOx stone disease [77]. As an omnivorous species, pigs share several anatomical and physiological characteristics with humans, including aspects of gastrointestinal anatomy, intestinal transit, renal architecture, and body size. Their multipapillary kidneys and similar renal handling of electrolytes and metabolites also provide an important setting that may be useful for investigating ductal processes that are difficult to study in commonly used rodent models [77,78,79].

The multipapillary architecture of porcine kidneys may be particularly relevant to studies of CaOx stone disease because it provides an anatomical framework for investigating papillary mineralisation, ductal plugging, and Randall’s plaque-associated mechanisms that are incompletely reproduced in rodents [17,80]. In addition, maximal urinary concentrating ability, renal haemodynamics, and glomerular filtration rate in pigs approximate human physiology more closely than those of mice or rats, facilitating the use of clinically relevant imaging modalities, endoscopic techniques, surgical procedures, and therapeutic interventions [81].

Compared with rodents, pigs are similar to humans with regards to their body size, renal anatomy, oxalate handling, and metabolic physiology. In addition, porcine models reproduce important aspects of human oxalate homeostasis, including hepatic metabolic pathways involving lactate dehydrogenase (LDH) and alanine-glyoxylate aminotransferase (AGXT), as well as Cl^−^/C_2_O_4_^2−^ transport mechanisms involving SLC26A6. These similarities may be related to oxalate homeostasis and CaOx deposition [80,82,83]. Furthermore, previous dietary studies in pigs have demonstrated CaOx accumulation at anatomical sites comparable to those affected in humans [78,80]. Outbred pig populations may also capture greater genetic variability than highly inbred laboratory rodent strains. These characteristics may be useful for investigating dietary hyperoxaluria, mechanisms of papillary crystal deposition, progression of calcium oxalate nephropathy, and preclinical evaluation of devices, surgical approaches, and emerging therapeutic strategies.

The potential advantages of the porcine model, however, are accompanied by practical limitations, including higher costs of housing and husbandry, specialised infrastructure, longer experimental timelines, and increased ethical and regulatory requirements. *Oxalobacter formigenes* colonization of swine caecum [84,85] represents another additional consideration when attempting to induce dietary hyperoxaluria using intact pigs. Despite these limitations, porcine models of hyperoxaluria and calcium oxalate urolithiasis can complement rodent and other experimental systems by providing a larger-animal setting for investigating selected aspects of disease pathogenesis and translation [80]. Their anatomical and physiological similarities to humans should therefore be considered in the context of specific research questions and should not be interpreted as evidence of universally superior translational or predictive validity.

### 8.2. Experimental Induction of Porcine Hyperoxaluria

Hyperoxaluria in pigs can be induced using pharmacological, dietary, or disease-based approaches, each reproducing different aspects of human oxalate metabolism. Dietary protein enrichment and HP supplementation increase endogenous oxalate production and promote CaOx papillary deposits considered potential precursors of stone formation, whereas controlled sodium oxalate administration provides a reproducible method for inducing acute or chronic hyperoxaluria under well-defined experimental conditions [79,81]. In studies by Mandel et al. [78] it was shown that feeding pigs with a 10% HP-enriched diet results in hyperoxaluria, calcium oxalate crystalluria (with maximal levels reached six days after feeding initiation), and calcium oxalate papillary deposits. The study includes an intriguing observation about the absence of a further increase in the response even after increasing the dietary content of HP up to 20%. Kaplon [86] and Patel [87] succeeded to induce hyperoxaluria in adult gravid sows with different concentrations of HP (10% and 5–10%, respectively, combined with various diets). Despite the success in the hyperoxaluria development, no calcium oxalate crystalluria or tissue deposits were reported in either study. Sivalingam et al. [88] reported the development of hyperoxaluria in gilts after 21 days of dietary 5% HP supplementation, along with the development of crystalluria and calcium oxalate deposits in renal tubules, collecting ducts, as well nephrolithiasis in papillary tips. Similarly, a diet containing 4% HP induced nephrocalcinosis in pigs and has been successfully used as a preclinical platform for evaluating oral oxalate-degrading enzyme therapy [89].

The sensitivity and relevance of porcine models was also previously shown in a pig model of secondary hyperoxaluria, induced by a high oxalate diet, made of rhubarb-enriched regular pig chow, with the calcium and magnesium content changed to reflect those of a Western diet [90]. The rhubarb-enriched diet was well tolerated by the pigs and led to a significant increase in urinary oxalate excretion levels, which were sensitive to oral oxalate-degrading enzyme therapy.

Penniston et al. [80] attempted to estimate the degree of oxaluria and CaOx crystal deposition in kidneys of Large White X Landrace gilts fed either high oxalate-high calcium, high oxalate-low calcium or normal oxalate-low calcium feed for 14 days. The authors observed hyperoxaluria in both groups of animals fed high oxalate, independent of the dietary Ca content, as well as the accumulation of ubiquitous and multiphasic calcium oxalate crystals and other histopathological signs of kidney injury. However, the authors used a very high dietary oxalate load in the diets of the pigs, which could possibly induce a toxic and non-physiological response.

A clinically relevant alternative to the aforementioned hyperoxaluria models is the porcine model of exocrine pancreatic insufficiency (EPI). Pancreatic enzyme deficiency results in fat malabsorption and dramatic changes in intestinal microbiome [91], thereby increasing intestinal oxalate absorption. When EPI pigs receive an oxalate-enriched diet, they develop hyperoxaluria and calcium oxalate nephrolithiasis, closely reproducing the pathophysiology of enteric hyperoxaluria observed in patients with pancreatic disease or other malabsorptive disorders [92,93]. This model also provides an opportunity to investigate pancreatic enzyme replacement therapy and other interventions targeting intestinal oxalate absorption.

Intravenous sodium oxalate (NaOx) infusion represents a complementary approach that isolates the renal consequences of systemic oxalate exposure by bypassing intestinal absorption and hepatic metabolism. In a recent porcine pilot study, graded 1% NaOx infusions administered over 15 h produced dose-dependent increases in plasma oxalate concentrations, together with transient hyperoxaluria that resolved within several hours after infusion ceased [81]. Repeated administration over 7–11 days resulted in sustained hyperoxalemia, progressive nephrocalcinosis, impaired renal function, and dose-dependent calcium oxalate deposition within renal tissue [81]. This dual protocol therefore spans both acute and chronic disease states: the acute phase is suitable for pharmacokinetic and dose-escalation studies, whereas the chronic phase provides reproducible histological and functional endpoints for therapeutic evaluation. Its principal limitation is that parenteral oxalate administration bypasses intestinal oxalate handling and therefore cannot be used to investigate gut-directed therapeutic strategies.

### 8.3. Emerging Human-Relevant Dietary Approaches

An important objective in current porcine research is to increase the physiological relevance of dietary hyperoxaluria models. Conventional diets supplemented with defined soluble oxalate salts provide precise control of exposure and high experimental reproducibility, but they do not reproduce the complex interactions among food composition, oxalate solubility, dietary calcium, magnesium, fiber, and intestinal processing that determine oxalate bioavailability in humans.

Whole-food dietary approaches may therefore provide complementary models of sustained dietary oxalate exposure. Such models have the potential to incorporate the food matrix and interactions between oxalate and other dietary components, thereby improving dietary realism. Their major limitation is reduced standardisation, because the oxalate content and bioavailability of plant-derived ingredients may vary according to cultivar, cultivation conditions, processing, and preparation.

Future dietary models should therefore combine improved physiological relevance with rigorous analytical characterisation of the final experimental diet. In particular, soluble and total oxalate concentrations should be measured directly, and the calcium-to-oxalate ratio, magnesium content, and other dietary factors influencing oxalate absorption should be reported. Such standardisation will be essential for determining whether more physiologically representative dietary paradigms improve the ability of experimental models to reproduce sustained hyperoxaluria and clinically relevant calcium oxalate-associated phenotypes.

### 8.4. Translational Applications

Porcine models may provide a useful intermediate experimental platform for evaluating selected dietary interventions, oxalate-binding compounds, oxalate-degrading enzymes, microbiome-directed therapies, endo-urological technologies, and other interventions that depend on large-animal anatomy or gastrointestinal physiology, before clinical investigation. The model is particularly well suited for assessing gut-directed therapies, including oral oxalate decarboxylase and engineered probiotic approaches, whose efficacy depends on intestinal luminal conditions that cannot be faithfully reproduced in vitro or adequately modelled in rodents alone. However, their principal value lies in addressing questions that cannot be adequately answered using small-animal systems alone.

**Figure 2:**
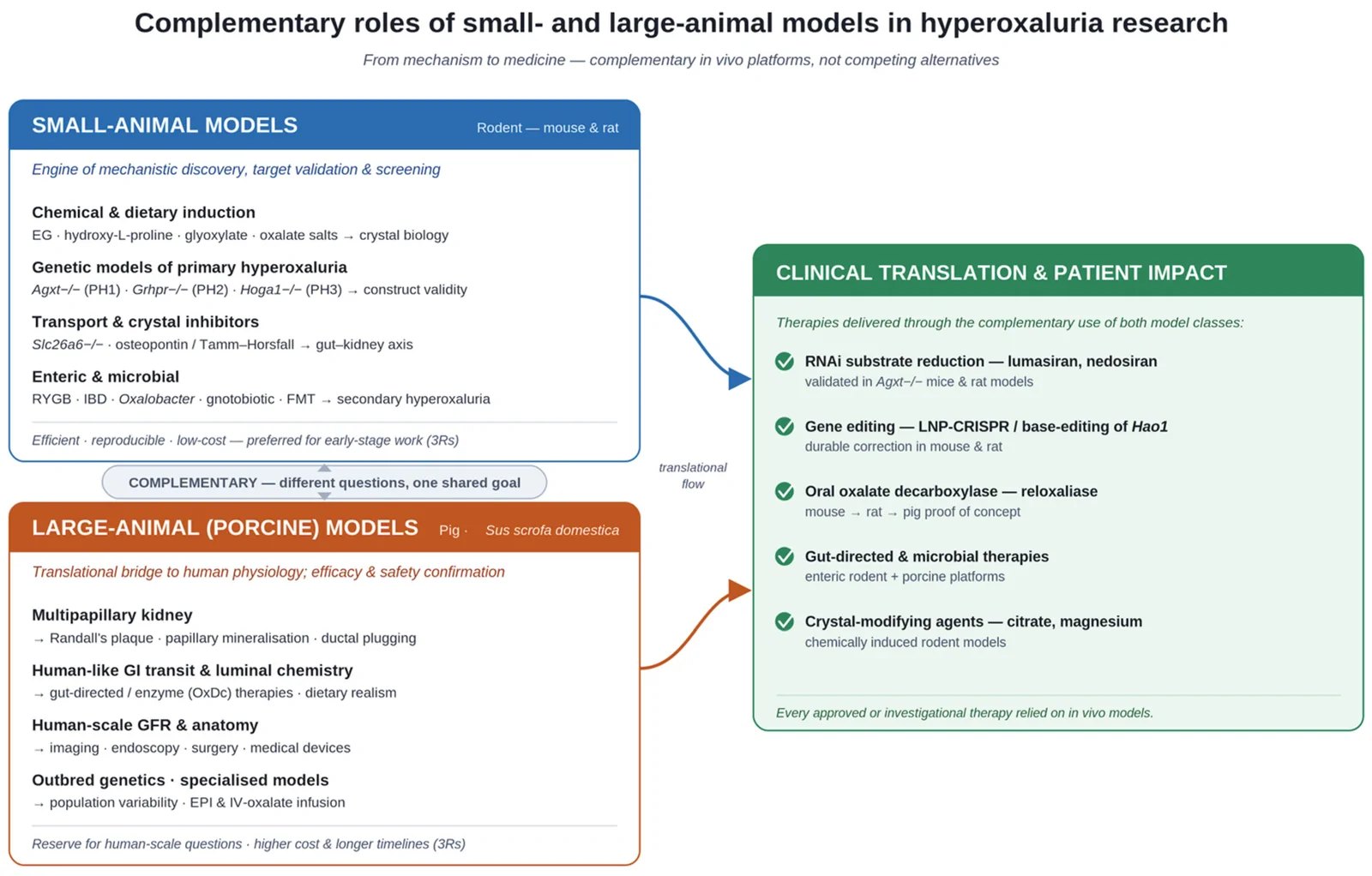
Complementary roles of small- and large-animal models in hyperoxaluria research. Small-animal models support mechanistic discovery, target validation, and early therapeutic screening, whereas large-animal models, particularly porcine models, provide a translational bridge to human physiology and clinically relevant efficacy and safety assessment. Complementary use of both model classes facilitates progression from mechanistic studies to translational development and clinical application.

Models may therefore be used sequentially (Figure 2) and rather than replacing rodent or genetic models, porcine models should be viewed as complementary translational platforms that bridge mechanistic discoveries from small-animal studies with aspects of human physiology. Rodent and genetic systems can support mechanistic discovery, target validation, and early therapeutic optimization, whereas porcine models may be used selectively to investigate questions requiring large-animal anatomy, clinically relevant procedures, or gastrointestinal and dietary conditions that cannot be adequately reproduced in smaller species. Their greatest value lies in evaluating therapeutic efficacy and safety under anatomical, physiological, and dietary conditions that more closely resemble those encountered in clinical practice.

## 9. Translational Applications of In Vivo Hyperoxaluria Models

The greatest translational value of experimental hyperoxaluria models lies in their contribution to therapeutic development. Virtually every mechanism-based therapy currently approved or under clinical investigation has relied on in vivo models for target validation, efficacy testing, dose optimisation, and preclinical safety assessment [94]. Different experimental systems have addressed complementary aspects of this process, with genetic models proving particularly valuable for substrate-reduction and gene-directed therapies, chemically induced models supporting evaluation of crystal-modifying agents, and enteric and large-animal models facilitating development of gut-directed interventions.

### 9.1. Substrate-Reduction RNA Interference

Genetic models of PH1 provided the essential proof of concept for hepatic substrate reduction by RNA interference (RNAi). Lumasiran, an N-acetylgalactosamine-conjugated small interfering RNA targeting *HAO1* (glycolate oxidase), was validated preclinically in *Agxt*-deficient mice and rat models of hyperoxaluria, where its precursor reduced urinary oxalate by up to 50% following a single administration and by approximately 98% after repeated dosing [95]. These studies established hepatic glycolate oxidase inhibition as a viable therapeutic strategy and directly supported subsequent clinical development, culminating in regulatory approval of lumasiran for the treatment of PH1 [96].

A similar translational pathway has been followed by nedosiran, which targets hepatic lactate dehydrogenase A (LDHA), the final common enzyme in oxalate synthesis. Evaluation in genetic mouse models of PH and in non-human primates demonstrated sustained oxalate lowering and supported the rationale for activity across PH1–PH3 [97]. Clinical translation closely mirrored these preclinical observations: lumasiran reduced 24-h urinary oxalate by approximately 54% compared with placebo in the pivotal ILLUMINATE-A trial, with subsequent studies demonstrating efficacy in infants and young children (ILLUMINATE-B) and in patients with impaired renal function (ILLUMINATE-C) [96,98,99]. Likewise, nedosiran achieved its primary urinary oxalate endpoint in the randomised PHYOX2 trial involving patients with PH1 and PH2 [100]. Together, these programmes represent one of the clearest examples of successful translation from genetic animal models to clinical practice.

### 9.2. Gene Therapy and Gene Editing

The same genetic models have served as the principal platforms for evaluating gene replacement and genome-editing strategies. Early studies demonstrated that AAV-mediated restoration of hepatic AGT expression corrected the biochemical phenotype of *Agxt*-deficient mice [38,39]. More recently, genome-editing approaches have shifted towards inactivation of upstream metabolic targets, particularly *Hao1*. LNP-mediated delivery of CRISPR–Cas9 targeting *Hao1* produced durable metabolic correction for up to 12 months in PH1 mice, while LNP-mediated base editing achieved sustained efficacy in rat models [44,45]. These experimental platforms continue to provide the principal systems for evaluating long-term efficacy, durability of gene editing, and potential off-target effects before clinical translation. Comprehensive reviews of gene- and RNA-based therapies for primary hyperoxaluria are available elsewhere [101].

### 9.3. Small-Molecule Substrate Reduction

Chemically induced and genetic hyperoxaluria models have also enabled validation of small-molecule approaches targeting endogenous oxalate synthesis. Stiripentol, an established antiepileptic agent, inhibits hepatic LDHA and has been shown to reduce oxalate production in hepatocytes, lower urinary oxalate excretion in vivo, protect against calcium oxalate nephrolithiasis and ethylene glycol toxicity in experimental animals, and reduce urinary oxalate in early clinical studies [102]. Similarly, inhibition of HYPDH using N-propargylglycine prevented nephrolithiasis, preserved renal function, and restored survival in the severe *Grhpr*-deficient PH2 mouse model [42]. These studies illustrate how experimental models enable both mechanistic target validation and evaluation of drug repurposing strategies before translation to patients.

### 9.4. Crystal-Modifying Therapies

Not all therapeutic approaches target oxalate synthesis directly. Rodent and large-animal models have been extensively used to evaluate interventions that inhibit crystal nucleation, growth, aggregation, or retention after hyperoxaluria has developed. Potassium citrate, magnesium supplementation, and other urinary crystallization inhibitors consistently reduce crystal burden in chemically induced hyperoxaluria models and remain important components of clinical stone prevention [11,16]. Unlike substrate-reduction therapies, these interventions require models that reliably reproduce crystal formation and renal deposition rather than oxalate overproduction alone. Accordingly, induced rodent models remain particularly valuable for investigating crystal biology and evaluating therapies that modify urinary crystallization.

### 9.5. Gut-Directed and Microbial Therapies

Models of enteric hyperoxaluria, transporter-deficient mice, and large-animal models have established the intestine as an attractive therapeutic target in both enteric and primary hyperoxaluria. Colonisation with *Oxalobacter formigenes* lowers urinary oxalate in rodent models through luminal oxalate degradation and stimulation of enteric oxalate secretion [63,64]. Although clinical studies, including the Oxabact (OC5) programme, have produced mixed results regarding urinary and plasma oxalate reduction, an acceptable safety profile has been consistently demonstrated [103,104,105]. Various lactic acid bacteria have likewise reduced urinary oxalate in experimental rodents [106,107], although clinical efficacy has generally been modest and strain dependent [108].

Experimental models have also been fundamental for evaluating enzyme-based therapies that directly degrade intestinal oxalate. Because the efficacy of these interventions depends on gastrointestinal physiology, intestinal transit, and luminal oxalate availability, both enteric rodent models and porcine models provide important complementary platforms for preclinical evaluation. Oral oxalate decarboxylase, discussed in detail in the following section, represents the most advanced example of this therapeutic strategy.

Collectively, these therapeutic programmes demonstrate that successful translation in hyperoxaluria has relied not on a single experimental model but on the complementary use of chemically induced, genetic, enteric, and large-animal systems. Each model contributes distinct mechanistic and translational information, and together they have enabled the rapid evolution of hyperoxaluria from a disorder with limited treatment options to one supported by multiple mechanism-based therapeutic strategies.

## 10. Oxalate Decarboxylase Therapy: Lessons from Animal Models

Oxalate decarboxylase (OxDc) represents the most extensively investigated enzyme-based strategy for gut-directed treatment of hyperoxaluria. Because the enzyme degrades luminal oxalate irrespective of its origin, it has potential applications across dietary, enteric, and PH. The development of OxDc-based therapies has relied heavily on complementary animal models, ranging from genetic mouse models to rats and pigs, each providing distinct mechanistic and translational insights.

### 10.1. Enzyme Biology and Therapeutic Rationale

Oxalate decarboxylase (EC 4.1.1.2), typified by the manganese- and oxygen-dependent YvrK/OxdC enzyme from *Bacillus subtilis*, catalyses the conversion of oxalate into formate and carbon dioxide. Degradation of oxalate within the intestinal lumen reduces the pool available for absorption and may promote net enteric oxalate secretion by maintaining a favourable concentration gradient between blood and the intestinal lumen, a mechanism analogous to that described for *Oxalobacter formigenes* [63]. Because this mechanism is independent of hepatic oxalate production, OxDc is theoretically applicable to both primary and secondary forms of hyperoxaluria.

The principal challenge is pharmaceutical rather than biological, namely maintaining enzymatic activity throughout the acidic and protease-rich gastrointestinal tract. Animal models have therefore been instrumental in evaluating three complementary delivery strategies: purified oral enzyme formulations, engineered probiotic bacteria expressing OxDc, and spore-forming bacterial platforms capable of delivering enzymatic activity within the gastrointestinal tract.

### 10.2. Oral Oxalate Decarboxylase Enzyme Therapy

Rodent and porcine models have established proof of concept for oral OxDc therapy. Cross-linked oxalate decarboxylase enzyme crystals (OxDC-CLEC) and related formulations reduced urinary oxalate excretion and prevented nephrocalcinosis in murine models of hyperoxaluria, including *Agxt*-deficient mice, demonstrating that degradation of intestinal oxalate can reduce systemic oxalate burden even when hepatic oxalate overproduction remains unchanged [109].

Subsequent studies in chronically hyperoxaluric rats demonstrated sustained reductions in urinary oxalate following oral OxDc administration, while porcine models extended these findings to a large-animal system. In pigs with HP-induced hyperoxaluria and nephrocalcinosis, prophylactic administration of OxDc effectively prevented the rise in urinary oxalate, whereas administration after hyperoxaluria had become established produced a more limited response [89]. These findings highlighted an important translational principle: therapeutic efficacy depends not only on enzymatic activity but also on formulation, timing of administration, intestinal pH, and gastrointestinal conditions, and the magnitude and source of oxalate exposure.

Collectively, studies in mice, rats, and pigs provided the pharmacodynamic foundation for development of the recombinant *Bacillus subtilis* OxDc preparation reloxaliase (ALLN-177). Subsequent clinical studies confirmed that reloxaliase reduced urinary oxalate excretion in patients with enteric hyperoxaluria, including the randomised URIROX-1 trial, thereby providing a clear example of successful translation from complementary animal models to human studies [110,111,112]. This translational pathway illustrates the value of using complementary experimental systems to address different components of therapeutic development rather than relying on a single model to establish efficacy.

### 10.3. Engineered Oxalate-Degrading Probiotics

Animal models have also enabled evaluation of living bacterial platforms designed to provide continuous OxDc production within the gastrointestinal tract. Recombinant *Lactobacillus plantarum* expressing and secreting heterologous *Bacillus subtilis* OxDc prevented renal calcium oxalate crystal deposition in hyperoxaluric rats, with secretory constructs consistently outperforming intracellular expression systems [113]. These studies demonstrated that sustained luminal enzyme delivery, rather than bacterial colonisation alone, is responsible for therapeutic benefit.

More recently, synthetic biology has extended this concept through engineered oxalate-consuming *Escherichia coli* strains that lowered urinary oxalate in experimental enteric hyperoxaluria models and showed predicted efficacy across different intestinal microbial communities [114]. Such approaches aim to overcome the variable intestinal colonisation that has limited the therapeutic use of native *Oxalobacter formigenes*.

### 10.4. Spore-Forming Probiotics

Spore-forming bacteria offer practical advantages as potential therapeutic platforms because spores can survive gastric transit and prolonged storage. *Bacillus subtilis*, which naturally expresses oxalate decarboxylase, therefore represents an attractive therapeutic candidate for gut-directed oxalate degradation. Experimental evidence, however, remains limited.

The strongest published support currently derives from a *Drosophila melanogaster* urolithiasis model, in which an oxalate-degrading *B. subtilis* strain reduced stone burden and improved survival [115]. Comparable mammalian studies remain scarce. Consequently, the therapeutic potential of spore-forming probiotic platforms should currently be regarded as promising but insufficiently established.

Future studies should determine the relationship between administered bacterial dose, intestinal survival, oxalate load, enzyme expression, and the resulting reduction in systemic oxalate burden. Evaluation in mammalian models representing dietary, enteric, and genetically determined hyperoxaluria will be necessary before the translational potential of these approaches can be defined more clearly.

### 10.5. Lessons for Model-Guided Development

Collectively, experimental studies demonstrate that successful OxDc therapy depends on considerably more than enzymatic activity alone. Animal models have shown that therapeutic efficacy is determined by interactions among enzyme delivery, gastrointestinal pH, intestinal transit, formulation, dietary composition, microbiome characteristics, and the magnitude and source of the oxalate exposure. Consequently, the choice of experimental model should reflect the intended clinical indication, whether dietary hyperoxaluria, enteric hyperoxaluria, or primary hyperoxaluria with markedly elevated endogenous oxalate production.

These studies also illustrate the complementary value of different experimental systems. Rodent and genetic models provide efficient platforms for mechanistic investigation and therapeutic optimisation, whereas large-animal models may be particularly informative for specific questions related to gastrointestinal physiology, dietary exposure, luminal chemistry, intestinal transit, and clinically relevant procedures. Together, these complementary animal models have contributed to establishing proof of concept, identifying the limitations of enzyme-based therapy, and guiding the translational development of OxDc from experimental studies to clinical evaluation. However, the available evidence does not support the concept of a universally optimal model; rather, confidence in translational findings is strengthened when therapeutic effects are reproduced across systems that address different biological and physiological aspects of oxalate homeostasis.

## 11. Cross-Cutting Considerations and Limitations

Several overarching considerations apply across all experimental models of hyperoxaluria and should inform both model selection and interpretation of findings.

Firstly, no currently available model fully reproduces the complexity of human disease [77]. Chemically induced models reliably generate hyperoxaluria and crystal deposition but generally represent acute or subacute processes that do not capture the decades-long natural history of idiopathic nephrolithiasis, including Randall’s plaque formation and ductal plugging [15,17,18]. Conversely, genetic models offer excellent construct validity by reproducing the underlying molecular defect, yet their phenotype may diverge from human disease because of species-specific differences in oxalate metabolism, as illustrated by the normoxaluric *Hoga1*-deficient mouse [43].

Secondly, reproducibility and comparability depend on rigorous experimental design and transparent reporting. Dietary composition—including calcium, oxalate, magnesium, and vitamin content—together with strain, sex, age, housing conditions, and renal maturity of the animals used, all substantially influence urinary oxalate excretion, crystal formation, and renal injury [22]. Consistent reporting of these variables, together with standardised outcome measures and normalisation of urinary oxalate to creatinine where appropriate, is essential for meaningful comparison between studies and for improving the reproducibility of preclinical research.

Thirdly, ethical considerations and the principles of the 3Rs (replacement, reduction, and refinement) should guide model selection. Severe models associated with marked morbidity or early mortality, such as hydroxy-L-proline-challenged *Grhpr*-deficient mice, require clear scientific justification, careful monitoring, and predefined humane endpoints. Simpler systems, including invertebrate models and other lower-complexity experimental platforms, should be preferentially used for early-stage mechanistic studies and therapeutic screening, whereas large-animal models should be reserved for questions that specifically require human-like anatomy, gastrointestinal physiology, or translational evaluation of medical devices and advanced therapies.

Ultimately, no single experimental system is universally applicable. The greatest translational value is achieved by selecting the model that best addresses the specific biological question while balancing construct validity, phenotypic relevance, reproducibility, ethical considerations, and clinical applicability.

## 12. Conclusions and Future Directions

In vivo models have contributed significantly to major mechanistic and therapeutic advances in hyperoxaluria research, from establishing the role of intestinal oxalate handling and the gut microbiome to enabling the development of RNA interference therapies now approved for PH. Their greatest strength lies not in the ability of any single system to reproduce the entire clinical disease spectrum, but in the complementary information provided by models that reproduce different mechanisms and disease phenotypes.

A central conclusion of this review is that hyperoxaluria, crystalluria, nephrocalcinosis, oxalate nephropathy, and nephrolithiasis should be distinguished when evaluating model validity. An experimental system that accurately reproduces one of these phenotypes may be highly informative for a specific biological question while having limited relevance for another. Genetic models faithfully reproduce the molecular basis of PH and provide indispensable platforms for gene- and RNA-directed therapies; chemically induced rodent models remain the cornerstone for investigating crystal biology, nephrocalcinosis, and acute or subacute oxalate-induced renal injury; enteric and microbiome-based models have revealed the importance of the gut–kidney axis, enable investigation of intestinal oxalate handling, and secondary hyperoxaluria; naturally occurring disease in companion animals may provide complementary insight into spontaneous and heterogeneous stone disease; and large-animal models provide an essential translational bridge by reproducing human gastrointestinal physiology, renal anatomy, dietary exposure, and clinically relevant interventions. Consequently, the most informative experimental strategy is not to identify a universally superior model, but to select and combine complementary models according to the specific biological or translational question being addressed.

Future progress will depend on the continued refinement of phenotype-oriented experimental systems. Important priorities include the development of models that better reproduce the slow progression and papillary pathology of idiopathic calcium oxalate stone disease, improved incorporation of microbiome-related mechanisms, and harmonization of experimental outcome measures and reporting standards to improve reproducibility. The value of large-animal systems should be established according to the specific questions they address, with particular attention to situations in which their anatomical or physiological characteristics provide information not available from smaller experimental models. Particular attention should be given to gut-directed approaches, including oxalate decarboxylase-based enzyme therapy and engineered microbial platforms, which complement hepatic substrate-reduction strategies by targeting intestinal oxalate, independently of its source.

Ultimately, the most informative translational strategy is not to identify a universally superior animal model, but to select and, where appropriate, combine complementary systems according to the specific mechanism, phenotype, or therapeutic intervention under investigation. Rigorous study design, transparent reporting, independent validation, and adherence to the principles of the 3Rs will be essential for improving reproducibility and strengthening the translation of experimental findings into effective and durable therapies for patients with hyperoxaluria. Together, these advances can improve the reliability and translational relevance of animal models, helping to bridge the gap between experimental research and clinical application in hyperoxaluria.

## Figures and Tables

**Table 1:** Comparative overview of currently available in vivo models of hyperoxaluria and calcium oxalate nephrolithiasis.

Model	Species	Strain, Sex, and Age Effects	Induction or Genetic Lesion	Principal Phenotype	Primary Outcome Measures	Main Strengths	Main Limitations
Ethylene glycol (EG)	Rat, mouse	Wistar > Fischer 344 for renal CaOx, driven by urinary macromolecule composition; intact males > females and castrated males; NH_4_Cl co-treatment amplifies strain differences	0.5–1.0% EG in drinking water ± NH_4_Cl	Hyperoxaluria; crystalluria; nephrocalcinosis with intratubular CaOx deposition; oxalate nephropathy (tubular injury, oxidative stress). Macroscopic stone formation is variable and generally less consistently reproduced than hyperoxaluria and renal CaOx deposition	Urinary oxalate (UOx); crystalluria; renal histology; inflammatory and oxidative-stress markers	Simple, reproducible, inexpensive; widely used for therapeutic screening	EG metabolites may cause direct nephrotoxicity; acidosis and strain/sex effects confound interpretation
Hydroxy-L-proline (HP)	Rat, mouse, pig	Strain-dependent oxalate yield in rats and mice; male animals predominantly used and sex effects not systematically compared; response scales with dietary HP concentration	3–10% dietary HP (4% in pigs)	Increased endogenous oxalate production; crystalluria and variable intrarenal CaOx deposition, depending on exposure conditions	UOx; crystalluria; renal histology; renal function	Physiological precursor; less nephrotoxic than EG; dose-tunable	Additional metabolic intermediates may contribute to phenotype; strain-dependent response
Soluble oxalate salts	Rat, mouse, pig	Outcome dominated by dose, route, duration of exposure and dietary calcium; strain and sex effects not systematically compared across studies	Acute sodium oxalate injection; dietary potassium oxalate; oxalate-rich low-calcium diet	Acute oxalate overload with crystalluria and acute crystal nephropathy, or, in chronic dietary protocols, nephrocalcinosis and oxalate-associated CKD	UOx; plasma oxalate; crystal burden; GFR; fibrosis; FGF23 and CKD markers	Precise control of oxalate exposure; models both acute injury and chronic oxalate nephropathy	Acute models poorly reproduce slow human stone formation
Whole-food high-oxalate diet	Pig, rat, mouse	Outcome governed by the dietary calcium-to-oxalate ratio and the food matrix; growth stage affects UOx/creatinine and requires normalization	Oxalate-rich whole-food diets ± altered dietary calcium	Dietary hyperoxaluria with variable crystalluria and renal CaOx deposition	UOx/creatinine; dietary oxalate intake; calcium-to-oxalate ratio; crystal deposition	Greater physiological relevance; incorporates food matrix and calcium interaction	Variable oxalate content of plant foods; reduced experimental reproducibility
Glyoxylate	Mouse	Background strain influences crystal burden in mice; male mice used almost exclusively; young adult animals standard	Repeated intraperitoneal glyoxylate administration	Rapid oxalate overproduction; tubular mitochondrial injury; CaOx crystalluria and intrarenal CaOx deposition; acute crystal-induced renal injury; nephrolithiasis can be reproduced under appropriate conditions.	Crystal deposition; oxidative stress; inflammatory pathways	Highly reproducible; ideal for early crystal–cell interaction studies	Acute model; limited relevance to chronic disease progression
*Agxt*−/− (PH1)	Mouse	Urolithiasis mainly in males; penetrance depends on background strain and degree of backcrossing, so crystal burden varies for the same allele	Targeted *Agxt* knockout ± EG challenge	Genetic hyperoxaluria and glycolate accumulation; variable crystalluria and nephrocalcinosis. Severe renal disease often requires an additional oxalate challenge.	UOx; glycolate; nephrocalcinosis; stone burden	Highest construct validity for PH1; major platform for RNAi and gene therapy studies	Mild spontaneous renal phenotype; often requires oxalate challenge
*Grhpr*−/− (PH2)	Mouse	Severity depends on HP dose and duration rather than on strain; sex effects not systematically reported	Targeted *Grhpr* knockout ± hydroxy-L-proline challenge	Severe hyperoxaluria; progressive nephrocalcinosis and oxalate nephropathy; CKD and reduced survival after HP challenge	UOx; plasma cystatin C; renal function; survival; crystal burden	Robust progressive phenotype suitable for therapeutic testing	Accelerated disease requires HP challenge
*Hoga1*−/− (PH3)	Mouse	Discrepancy with human PH3 is a species difference (persistent mitochondrial AGT activity in mice) rather than a strain effect	Targeted *Hoga1* knockout	Altered HP metabolism; predominantly biochemical phenotype; increased HOG/DHG metabolites, with limited renal phenotype in the unchallenged mouse	Urinary oxalate; HOG/DHG metabolites	Reproduces PH3 biochemical defect	Poor face validity for the renal phenotype because mice may remain normoxaluric
*Slc26a6*−/−	Mouse	Phenotype expression governed by dietary oxalate exposure; urolithiasis reported predominantly in males	Targeted deletion of intestinal Cl^−^/oxalate exchanger	Enteric hyperoxaluria; hyperoxalemia; CaOx urolithiasis resulting from altered intestinal oxalate transport	UOx; plasma oxalate; intestinal oxalate flux; stone formation	Defines intestinal oxalate transport and gut–kidney axis; strong human mechanistic relevance	Phenotype strongly influenced by dietary oxalate exposure
Osteopontin/Tamm–Horsfall protein knockout	Mouse	Single knockouts largely protected irrespective of sex; combined deficiency plus challenge required; background strain sets baseline inhibitor capacity	Single or combined deletion ± oxalate challenge	Impaired endogenous crystal inhibition; enhanced CaOx deposition and stone formation after challenge in combined deficiency	Crystal deposition; papillary calcification; urinary crystal inhibitors	Defines mechanisms of crystal nucleation, aggregation, and retention	Single knockouts show limited spontaneous phenotype; challenge required
Enteric/malabsorptive hyperoxaluria models	Mouse	Diet-induced obese and ileitis-prone (SAMP1/YitFc) backgrounds are not interchangeable; phenotype also varies with surgical success	RYGB surgery or inflammatory bowel disease models + high-fat oxalate-enriched diet	Fat malabsorption; increased intestinal oxalate absorption; hyperoxaluria. Crystal deposition variable; stones not consistently formed	UOx; faecal fat; microbiome composition; intestinal oxalate handling	Reproduces secondary hyperoxaluria and gut–kidney interactions	Technically demanding; variable phenotype
*Oxalobacter*/gnotobiotic/FMT models	Mouse, rat	Colonization efficiency differs between rats and mice and with donor microbiota; age at colonization matters; sex effects rarely reported	Colonization with *O. formigenes*; germ-free models; faecal microbiota transfer	Altered microbial oxalate degradation and intestinal secretion; modifies rather than induces the hyperoxaluric phenotype	UOx; intestinal oxalate flux; microbiome profiling	Dissects microbial regulation of oxalate homeostasis	Colonisation instability; species-specific differences
Porcine dietary and pharmacological models	Pig	Age (growing vs. adult) alters GFR and therefore UOx/creatinine; gravid sows and gilts give different results; breed line and sex seldom reported	Dietary or pharmacological oxalate loading (K-oxalate, HP, dietary approaches)	Sustained hyperoxaluria; crystalluria; papillary CaOx deposition; nephrolithiasis in papillary tips in some studies	UOx/creatinine; crystalluria; Yasue histochemistry; renal function	Human-like gastrointestinal and renal physiology; suitable for dietary and enzyme therapies	High cost; ethical constraints; age-related GFR changes
Porcine intravenous sodium oxalate infusion	Pig	Growing animals have unstable GFR; dose must be scaled to body weight; sex effects not reported	Acute (15 h) and repeated (7–11 d) intravenous sodium oxalate administration	Reversible acute hyperoxalemia; chronic nephrocalcinosis after repeated exposure	Plasma oxalate; UOx; renal CaOx area; renal function	Separates systemic oxalate toxicity from intestinal absorption; quantitative histological endpoint	Bypasses gut handling; currently limited to pilot-scale studies
Porcine exocrine pancreatic insufficiency model	Pig	Outcome depends on completeness of pancreatic duct ligation; age and growth stage affect UOx/creatinine	Pancreatic duct ligation + high-fat oxalate-enriched diet	Enteric hyperoxaluria; CaOx crystalluria and stone formation	UOx; faecal fat; crystal and stone burden	Clinically relevant model of malabsorption-associated hyperoxaluria; enables enzyme replacement studies	Surgical complexity; limited availability and published experience

Abbreviations: CaOx, calcium oxalate; CKD, chronic kidney disease; DHG, 2,5-dihydroxyglutarate; FGF23, fibroblast growth factor 23; FMT, fecal microbiota transplantation; GFR, glomerular filtration rate; HOG, 4-hydroxy-2-oxoglutarate; HP, hydroxy-L-proline; PH1–PH3, primary hyperoxaluria types 1–3; RYGB, Roux-en-Y gastric bypass; UOx, urinary oxalate.

## Data Availability

No new data were created or analyzed in this study. Data sharing is not applicable to this article.

## Abbreviations

The following abbreviations are used in this manuscript:

AAV	Adenoviral and adeno-associated viral vectors
AGT	Alanine-glyoxylate aminotransferase
CaOx	Calcium oxalate
CKD	Chronic kidney disease
DHG	2,5-dihydroxyglutarate
EG	Ethylene glycol
EPI	Exocrine pancreatic insufficiency
FMT	Faecal microbiota transplantation
FGF23	Fibroblast growth factor 23
GFR	Glomerular filtration rate
GRHPR	Glyoxylate/hydroxypyruvate reductase
HAO1	Hydroxy-2-oxoglutarate aldolase
HOG	4-hydroxy-2-oxoglutarate
HOGA1	Hydroxy-2-oxoglutarate aldolase
HP	Hydroxy-L-proline
HYPDH	Hydroxyproline dehydrogenase
LDHA	Hepatic lactate dehydrogenase A
LNP	Lipid nanoparticle
NaOx	Sodium oxalate
OxDc	Oxalate decarboxylase
PH1–PH3	Primary hyperoxaluria types 1–3
RNAi	RNA interference
UOx	Urinary oxalate
RYGB	Roux-en-Y gastric bypass
